# Impact of Oncotype DX Recurrence Score on Treatment Decisions: Results of a Prospective Multicenter Study in Turkey

**DOI:** 10.7759/cureus.522

**Published:** 2016-03-08

**Authors:** Vahit Ozmen, Ajlan Atasoy, Erhan Gokmen, Mustafa Ozdogan, Nilufer Guler, Cihan Uras, Engin Ok, Orhan Demircan, Abdurrahman Isikdogan, Pinar Saip

**Affiliations:** 1 Department of Surgery, Istanbul University; 2 Medical Department, EORTC; 3 Department of Internal Medicine, Division of Medical Oncology, Ege University; 4 Department of Internal Medicine, Division of Medical Oncology, Akdeniz University / Memorial Hospital Antalya; 5 Department of Internal Medicine, Division of Medical Oncology, Hacettepe University Institute of Oncology; 6 Department of General Surgery, Acibadem University Maslak; 7 Department of General Surgery, Erciyes University; 8 Department of General Surgery, Cukurova University / Acibadem University Adana; 9 Department of Internal Medicine, Division of Medical Oncology, Dicle University; 10 Department of Medicine, Division of Medical Oncology, Istanbul University

**Keywords:** breast cancer, adjuvant treatment, oncotype dx, decision impact, genomic testing

## Abstract

Introduction: Breast cancer is the most common malignancy among Turkish women and the rate of early stage disease is increasing. The Oncotype DX^®^ 21-gene assay is predictive of distant recurrence in ER-positive, HER2-negative early breast cancer. We aimed to evaluate the impact of the Recurrence Score^®^ (RS) on treatment decisions and physician perceptions in Turkey. We also studied correlations between RS and routine risk factors.

Patients and Methods: Ten academic centers across Turkey participated in this prospective trial. Consecutive breast cancer patients with pT1-3, pN0-N1mic, ER-positive, and HER2-negative tumors were identified at multidisciplinary tumor conferences. The initial treatment decision was recorded before tumor blocks were sent to the central laboratory. Each case was brought back to tumor conference after receiving the RS result. Both pre- and post-RS treatment decisions and physician perceptions were recorded on questionnaire forms. Correlations between RS and classical risk factors were evaluated using univariate and multivariate analyses.

Results: Ten centers enrolled a total of 165 patients. The median tumor size was 2 cm. Of 165 patients, 57% had low RS, 35% had intermediate RS, and 8% had high RS, respectively. The overall rate of change in treatment decision was 33%. Initially, chemotherapy followed by hormonal therapy (CT+HT) was recommended to 92 (56%) of all patients, which decreased to 61 (37%) patients post-RS assay (p<0.001). Multivariate analysis indicated that progesterone receptor (PR) and Ki-67 scores were significantly related to RS.

Conclusion: Oncotype DX testing may provide meaningful additional information in carefully selected patients.

## Introduction

Invasive breast carcinoma is the most commonly seen malignancy and the leading cause of cancer-related death in Turkish women, both in premenopausal and postmenopausal age groups. Due to the higher percentage of younger population in Turkey, 45% of all breast cancer patients are premenopausal [1]. The breast cancer incidence rate is gradually increasing in this country due to westernized lifestyle, population growth and aging, and most importantly, the successful implementation of nationwide opportunistic screening programs in newly opened cancer screening centers. The latter contributed to a higher proportion of earlier stage disease reported in recent decades [2]. According to the Turkish Ministry of Health, nearly half of all breast cancer cases across the country were diagnosed in early stage in 2011 [3]. A recent analysis of 13,240 patients in the National Breast Cancer Database established within Turkish Federation of Breast Diseases Societies showed that 50% of patients had pN0 disease, with 27% of all breast cancer patients diagnosed with Stage I disease. Overall, 62% of patients had pathologic characteristics of Luminal-A type breast cancer [1].

The prognostic features most commonly used in adjuvant treatment decision for node-negative patients include patient age, menopausal status, tumor size, tumor grade, Ki-67 score, HER2 status, and strength of estrogen receptor (ER)/progesterone receptor (PR) expression. While the treatment decision is easier for patients with unequivocal features, it becomes challenging to personalize therapy for those early stage breast cancer patients who have less clearly defined features, especially when they are young. Occasionally, agreeing on a treatment plan may be difficult in tumor conference even for tumors with “luminal A-like” phenotype, which are believed to be less responsive to chemotherapy [4-5].

With an increasing breast cancer incidence and with nearly half of new breast cancer cases presenting pN0 stage in Turkey, overtreatment is gaining significance as a health care and medical ethics issue facing Turkish physicians and patients, as well as the national health insurance system that provides extensive coverage for cancer treatment and treatment related toxicities.

There is increasing evidence that molecular tests may have a role in individualizing therapy. The Onco*type* DX^®^ 21-gene assay quantifies the likelihood of distant recurrence in women with ER-positive, lymph node-negative breast cancer treated with adjuvant tamoxifen, and it has been validated to predict benefit from chemotherapy in this population [6-7]. It has been incorporated into commonly accepted guidelines including the National Comprehensive Cancer Network (NCCN) [8], American Society of Clinical Oncology(ASCO) [9], European Society of Medical Oncology (ESMO) [10], and St. Gallen Consensus guidelines [4]. The analysis of women in the lowest risk group of the recently reported TAILORx trial (clinicaltrials.gov identifier NCT00310180) provided prospective evidence that this low risk group (Onco*type* DX^®^ Recurrence Score 0-10) may potentially be spared chemotherapy, with 5-year rates of distant relapse-free survival of 99%, invasive disease free survival of 94%, and of overall survival of 98% with hormonal therapy alone [11].

Onco*type* DX^®^ 21-gene assay is not widely considered feasible by Turkish physicians due to its cost, as it’s currently not reimbursed by the Turkish Social Security Administration. We designed a prospective multicenter study aiming to assess the impact of the Oncotype DX Recurrence Score result (RS) on treatment decisions and the physicians’ perceptions regarding influence of RS results on their final treatment recommendations. We also analyzed the correlation between RS and pathologic risk factors routinely used at our tumor conference discussions.

## Materials and methods

### Patients and study design

Ten academic centers in seven Turkish cities that routinely discuss all new breast cancer cases at weekly multidisciplinary tumor conferences participated in this prospective trial. The study was approved by a central Ethics Committee, as well as by each Institutional Review Board. Consecutive breast cancer patients with pT1-3, pN0-N1mic, M0, ER (+), and HER2 (-) tumors were identified, and adjuvant treatment decisions were made with careful consideration of clinical and pathologic information by all of the tumor conference members. This initial treatment decision (pre-RS assay decision) was recorded on a questionnaire form by site investigators, and baseline pathologic characteristics were recorded in an enrollment form. The patients identified at the tumor conference were individually contacted. The pre-RS assay decision was conveyed, and informed consent was obtained. Formalin Fixed-Paraffin Embedded (FFPE) tissue blocks were sent to the central laboratory (Genomic Health, Inc., California, USA). Cases were discussed at the tumor conference again when the RS became available and investigators filled the post-RS assay questionnaire forms with the final decision. Patients were notified of their score along with the final recommendation. The pre- and post-RS assay questionnaires also contained questions aimed to capture how strongly the investigator believed that the RS assay result would contribute and has contributed to the final treatment decision, respectively.

### Statistical analysis

Statistical analyses on Oncotype-DX RS were conducted using both nominal data based on the actual RS score and an ordinal scale with three RS categories (<18, 18-30, >30) as per standard practice. The change in the chemotherapy decision between pre- and post-RS assay treatment plans was analyzed using McNemar’s test. Associations between RS categories (<18, 18-30, >30) and clinicopathologic parameters were evaluated using Mantel-Haenszel Chi-Square test. Integrated evaluation by multivariable analysis was carried out to study the association between RS (dependent variable) and all clinicopathologic risk factors (predictors) using linear regression models. The risk factors (independent variables) included in the multivariable regression analysis were age, tumor size, tumor grade, ER score, PR score, Ki67 score and HER2 score (per immunohistochemistry). The cut-off for the p-value was taken as less than 0.05 for statistical significance in all analyses performed.

## Results

### Patient and tumor characteristics

In total, 165 patients were enrolled from ten centers across Turkey. Median age was 49 years (range=26-76). Table 1 outlines the patient and tumor characteristics at the time of surgery. 108(65.5%) patients had pT1 tumors, and the median tumor size was 2 cm (range=0.6-8.0cm). Only 11(6.7%) patients had micrometastasis in axillary lymph nodes (pN1mic). The majority of patients had Modified Scarff Bloom Richardson Grade 2 tumors (n=108, 65.5%). Overall, 76 (53.5%) patients had a Ki-67 score of <20%, including 60 patients whose Ki-67 scores were less than 14%. Based on PR and Ki-67 scores, 90 (60.4%) patients were considered to have characteristics of luminal B molecular subtype.

**Table 1:** Patient and tumor characteristics (n=165) “Luminal subtypes” were defined based on PR and Ki67 evaluation as follows: Luminal-A=PR score ≥20 and Ki-67 <20%; Luminal-B=PR<20% or Ki67>20%. 16 patients had missing Ki67 data and therefore a subtype could not be assigned.

		n (%)
Age	<40 years	23 (14.0)
	40-49 years	70 (42.4)
	≥50 years	72 (43.6)
Tumor size	≤1 cm	19 (11.5)
	1-2 cm	89 (53.9)
	>2 cm	57 (34.5)
LN Status	pN0	154 (93.3)
	pN1mic	11 (6.7)
Grade	1	28 (17.0)
	2	108 (65.5)
	3	26 (15.8)
ER score	≤ 10%	6 (3.6)
	11-30%	4 (2.4)
	31-50%	6 (3.6)
	51-70%	14 (8.5)
	>%70	135 (81.8)
PR score	≤20%	54 (32.7)
	>%20	111 (67.3)
Ki67 score	<20%	76 (53.5)
	≥%20	66 (46.5)
Luminal Subtype (n=145)*	Luminal-A	59 (39.5)
	Luminal-B	90 (60.4)

### Oncotype DX® results and treatment decision

The mean Oncotype DX^®^ recurrence score (RS) was 18.8±14.0 (range=0-64; median RS=16). RS was low in 56.8%, intermediate in 35.2%, and high in 8.5% of patients, respectively.

**Figure 1:**
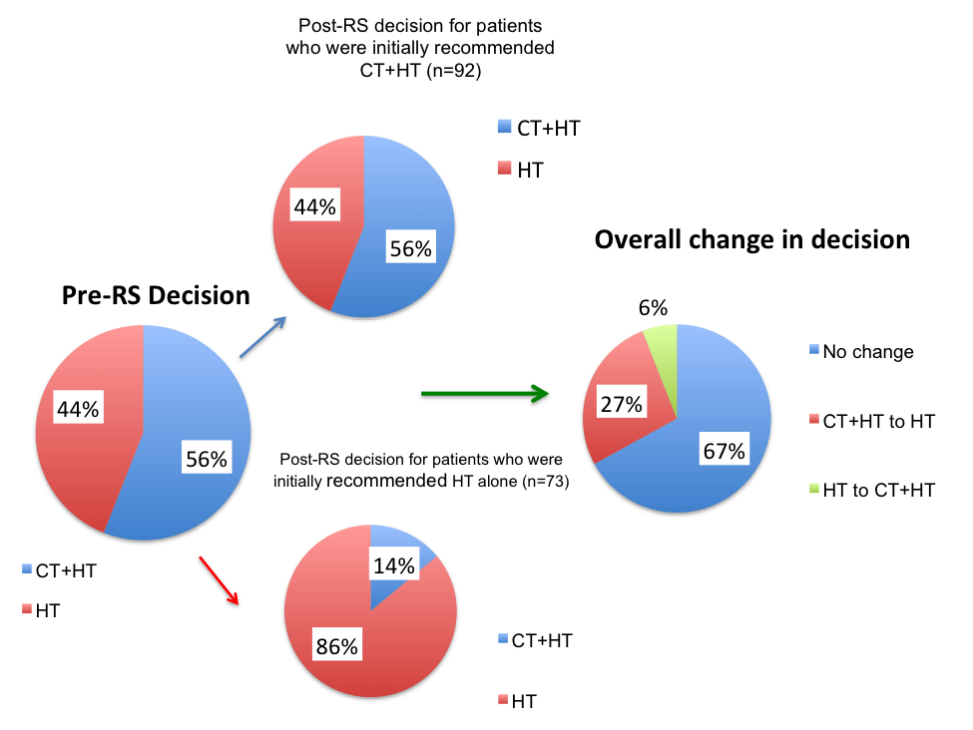
Change in Treatment Decision

The overall rate of change in treatment decision following RS result was 33% (Figure 1). Following initial tumor conference (pre-RS assay), 92 (55.8%) patients were recommended chemotherapy followed by hormonal therapy (CT+HT), (Table 2). There was a significant decrease in this number following the post-RS assay tumor conference; the final treatment recommendation was CT+HT in 61 (37.0%) patients (McNemar test, p<0.001). Almost half (45%) of the patients who were originally recommended CT+HT were recommended HT alone after receiving the RS results. Meanwhile, among those patients for whom the initial recommendation was HT alone (n=73), 10 (13.7%) were recommended CT+HT after the discussion of the RS results. Among the patients with low and intermediate scores, the decision changed from CT+HT to HT alone in 33(78.6%) and 8(20.5%) patients, respectively. In the intermediate RS group, 12 (63.2%) patients were recommended HT alone both at pre- and post-RS assay tumor conference. The initial treatment recommendation was HT alone for 3 of the 14 patients in the high-risk RS group. Following review of RS results, all 14 patients in this group were recommended CT+HT.

**Table 2:** Treatment decision in patients before and after RS, and the distribution of RS groups *McNemar test RS, risk score; HT, hormonal therapy; CT+HT, chemotherapy followed by hormonal therapy.

				Post-RS Decision		
				HT	CT+HT	
			n	n (%)	n (%)	p value*
Pre-RS Decision	All patients	HT	73	63 (86.3)	10 (13.7)	<0.001
		CT+HT	92	41 (44.6)	51 (55.4)	
		Total	165	104 (63.0)	61 (37.0)	
	Low RS	HT	51	51 (100.0)	0 (0.0)	
		CT+HT	42	33 (78.6)	9 (21.4)	
		Total	93	84 (90.3)	9 (9.7)	
	Intermediate RS	HT	19	12 (63.2)	7 (36.8)	
		CT+HT	39	8 (20.5)	31 (79.5)	
		Total	58	20 (34.5)	38 (65.5)	
	High RS	HT	3	0 (0.0)	3 (100.0)	
		CT+HT	11	0 (0.0)	11 (100.0)	
		Total	14	0 (0.0)	14 (100.0)	

### Physician perceptions

When the pre-assay questionnaires of all the cases with treatment decision changes were evaluated, it was noted that only 31.4% of investigators “strongly believe(d)” that Oncotype DX result would contribute to final treatment decision. This rate increased to 88.2% for this group in the post-assay questionnaires. For the same group of patients, 41.2% and 85% of investigators indicated they “strongly believe(d)” that the test would provide additional information in the pre-assay and post-assay questionnaires, respectively.

### Associations between RS and clinicopathologic features

When age groups were analyzed with three different cut-off values (age < or ≥ 40, 45, or 50 years), age was not found to be a significant predictor of RS in either univariate or multivariable analysis (Mantel-Haenszel test for univariate and regression model for multivariable analysis; table not included). Among patients who were younger than 40 years of age, 52.2% had a low RS, 30.4% had an intermediate RS, and only 17.4% had a high RS. For patients over age 50, these ratios were 54.8%, 31.0% and 12.1%, respectively.

In univariate analysis, grade (p=0.002), Ki-67 score (14% and 20% cut-offs; p<0.001 for both groups), and PR score (cut-off 20%; p<0.001) were the only risk factors that significantly correlated with RS, whereas tumor size, LN status (presence of micrometastasis), and ER score were not found to be significant predictors of RS (Mantel-Haenszel test; table not included). Among patients with luminal A-molecular subtype as per their PR and Ki-67 scores, the vast majority (81.4%) had a low RS, and only 1.7% had a high RS. In contrast, only 41.1% of patients with luminal B tumors had a low RS, and 13.3% had a high RS (Mantel-Haenszel test, p<0.001).

Multivariable analysis of all risk factors including Ki-67, age, tumor size, ER score, PR score, and HER2 score (0 vs +1) showed that the combination of all these numerical variables constituted a statistically significant regression model for predicting RS (R=0.671, R^2^=0.450, p<0.001). When each variable is examined, Ki-67 and PR score were the only variables that seemed to significantly contribute to estimating the RS (Table 3).

**Table 3:** Correlation between RS and clinicopathologic factors, multivariate regression analysis

R	R^2^	Corrected R^2^	SE	p value
0.671	0.450	0.421	7.961	<0.001
Variables	B	SE	Beta	p value
Fixed	23.066	4.816		<0.001
Ki-67 score (%)	0.286	0.047	0.424	<0.001
PR score (%)	-0.111	0.019	-0.381	<0.001
Age (years)	-0.104	0.066	-0.106	0.118
Grade	1.576	1.308	0.084	0.231
HER-2 (0 or +1)	0.985	0.937	0.069	0.295
Tumor size (cm)	-0.733	0.706	-0.068	0.301
ER score (%)	-0.027	0.030	-0.059	0.374

## Discussion

### Decision impact

Personalizing therapy for node-negative, early stage breast cancer patients presents an important challenge that Turkish physicians have been facing more frequently in the recent years, especially following the improvement of cancer screening programs. Due to the high cost of molecular profiling tests in general, it has been difficult to incorporate patients in treatment planning, and moreover, there is an ongoing debate within the oncology community with regard to what extent such tests impact treatment decisions. This study is the first prospectively designed multicenter trial in Turkey aimed to explore the decision impact of using Onco*type *DX. Our results showed that treatment decisions changed in nearly one-third of all cases, and more patients were recommended HT alone as a result of integrating RS assay in tumor conference (Figure 1). This decision impact rate is similar to some earlier studies performed in Western Europe. In a prospective German study including 366 assessable patients with ER(+), HER2(-) early breast cancer and 0–3 positive lymph nodes (244 patients with pN0 disease), treatment recommendations changed in 33% of all patients (pN0 30%, pN1 39%), and compared to overall pre-RS assay recommendations, 33% (pN0 29%, pN1 38%) fewer patients actually received chemotherapy in the end [12]. In a UK trial that included 142 patients with pN0 and pN1mic disease, treatment decision was changed for 26.8% of all women, including 26 (45.6%) of 57 patients who were spared chemotherapy [13]. More recently, a report from a retrospective cohort study in Ireland included the observation that the availability of RS assay was inversely related to the use of chemotherapy and that of those patients who underwent Oncotype DX testing, 57% were spared chemotherapy [14]. The higher change rate in the Irish cohort may potentially be due to a selection of patients for the assay compared to prospective studies where consecutive patients would be enrolled. Our findings contribute to the observation that molecular testing provides clinically meaningful additional information for a significant proportion of patients.

### Associations between RS and routine risk factors

While our results suggest a correlation with some pathologic features, the RS result made a significant impact in clinical practice despite rigorous pathologic evaluation at our academic centers. The significant predictors in our multivariate analysis included the Ki-67 score, which is also considered to be important in predicting luminal subtype. It should be noted that significant inter-laboratory variability is a notable concern when interpreting Ki-67 score, especially in Grade 2 tumors [15]. Our multivariable analysis also suggests that the PR score may have a predictive value in estimating the risk group, as also reported in earlier literature [16]. As in interpretation of the Ki-67 score, variability in immunohistochemistry results could potentially influence clinician confidence in PR score while planning treatment. The strength of PR expression may help identify those patients who could require a more careful evaluation of prognostic parameters, and potentially molecular testing.

Some groups have argued that results of a careful pathologic examination negate the need for Oncotype DX testing and routine pathologic parameters, or composite indexes created using these parameters can predict Oncotype DX assay result [17-20]. On the other hand, despite the predictive value of a careful pathologic evaluation, breast oncologists tend to overestimate the recurrence risk in a considerable number of patients [21]. The comfort level in sparing a patient from chemotherapy may be even lower in regions with less experience with utilizing molecular testing in routine practice. Moreover, patients with equivocal pathologic features, most of whom have luminal-B subtype tumors, may be conflicted about the treatment recommendation. According to their most recent consensus report, St. Gallen Expert Panel did not believe chemotherapy should be recommended in all patients with luminal B-like disease, and that it could be omitted in cases with low scores on Oncotype DX. 

## Conclusions

Oncotype DX may provide additional information to improve personalized therapy in a significant proportion of patients with early stage breast cancer. More frequent use in carefully selected patients may help spare patients from chemotherapy, and in some rare instances, it may help correctly identify high-risk patients who would otherwise be recommended hormonal therapy alone. Moreover, when used with careful consideration, it may increase level confidence in treatment recommendation. Among pathologic parameters, Ki-67 score and PR score seem to correlate with RS, which is expected as these parameters are included among the 16 cancer-related genes in the score.
